# Explainable DSC Perfusion Radiomics for Post-Treatment Glioblastoma Assessment Using a Soft-Voting Machine-Learning Ensemble

**DOI:** 10.3390/cancers18213567

**Published:** 2026-11-06

**Authors:** Rafail C. Christodoulou, Giorgos Christofi, Georgios Vamvouras, Mateus A. Esmeraldo, Platon S. Papageorgiou, Evros Vassiliou, Elena E. Solomou, Sokratis G. Papageorgiou, Michalis F. Georgiou

**Affiliations:** 1 Division of Neuroimaging and Neurointervention, Department of Radiology, Stanford University, Stanford, CA 94305, USA; rafail99@stanford.edu; 2 Faculty of Electrical Engineering, Mathematics and Computer Science, Delft University of Technology, 2628 CD Delft, The Netherlands; 3 Department of Electrical and Computer Engineering, National Technical University of Athens (NTUA), 15780 Athens, Greece; gvamvouras@mail.ntua.gr; 4 Department of Radiology, Stanford University, Stanford, CA 94305, USA; 5 Department of Medicine, National and Kapodistrian University of Athens, 11527 Athens, Greece; platopap@med.uoa.gr; 6 Department of Biological Sciences, Kean University, Union, NJ 07083, USA; 7 Internal Medicine-Hematology, University of Patras Medical School, 26500 Rion, Greece; 8 1st Department of Neurology, Eginition Hospital, Medical School, National and Kapodistrian University of Athens, 11528 Athens, Greece; sokpapa@med.uoa.gr; 9 Department of Radiology, University of Miami, Miami, FL 33136, USA

**Keywords:** glioblastoma, dynamic susceptibility contrast, perfusion MRI, radiomics, machine learning, ensemble learning, pseudoprogression, radiation necrosis, treatment response assessment, arterial spin labeling

## Abstract

Background/Objectives: After chemoradiation for glioblastoma, residual or recurrent tumor (tumor-positive disease, TP) and treatment-related change (TRC), which includes pseudoprogression and radiation necrosis, look alike on conventional MRI. We tested whether radiomic features from dynamic susceptibility contrast (DSC) perfusion MRI alone can separate them. Methods: We analyzed 224 post-treatment MRI examinations from 168 patients in the public UCSD-PTGBM collection. A machine-learning ensemble was trained on 1223 radiomic features from the leakage-corrected relative cerebral blood volume (rCBV) map, with training (109 patients, 143 examinations) and test (59 patients, 81 examinations) data separated at the patient level and the model fixed before testing. The analysis was repeated on the cerebral blood flow (CBF) map from the same scans, and DSC was compared with arterial spin labeling (ASL) in patients who had both. Results: On the independent test set (patient-level analysis), the model distinguished TP from TRC with an area under the receiver operating characteristic curve (AUC) of 0.79 (95% CI 0.67–0.89). It detected 31 of 49 patients with tumor (sensitivity 63%) and correctly identified all 10 patients with TRC (specificity 100%); at a predefined higher-sensitivity cut-off, sensitivity was 69% and specificity 80%. With the CBF map, the AUC did not differ significantly (0.78; *p* = 0.89), but there was a different balance of sensitivity and specificity. In an exploratory subset of 36 patients, DSC and ASL did not differ significantly (AUC 0.82 vs. 0.71; *p* = 0.52). Conclusions: DSC perfusion radiomics alone identified tumor with high specificity but missed about one-third of tumors: a positive result may support a diagnosis of tumor, whereas a negative result cannot exclude it. External, multicenter validation is needed before clinical use.

## Simple Summary

After treatment for glioblastoma, an aggressive brain tumor, follow-up MRI often cannot show whether an abnormality is returning tumor or a reaction of the brain to radiation and chemotherapy. This matters because returning tumor needs new treatment, whereas a treatment reaction can be watched. We tested whether a computer model reading only perfusion MRI, a widely available scan that measures blood volume inside the lesion, could tell the two apart. We used 224 follow-up scans from 168 patients in a public dataset; 173 scans (77%) showed tumor and 51 (23%) showed treatment-related change. In a separate test group of 59 patients (81 scans), the model correctly recognized all 10 patients with treatment-related change (specificity 100%) and 31 of the 49 patients with tumor (sensitivity 63%). A positive result therefore strongly suggested tumor, but a negative result could not rule it out. Accuracy did not differ significantly with a second type of perfusion map from the same scans, but the best decision cut-off changed, so hospitals using such tools need to report how their perfusion maps were produced.

## 1. Introduction

Glioblastoma (GBM) is the most aggressive primary brain tumor in adults. Despite aiming for maximal safe surgical resection followed by chemoradiation, the overall survival rate remains low [1,2,3]. The goal of this regimen is to improve outcomes, but it complicates post-treatment imaging surveillance because treatment-related changes (TRCs), which encompass radiation necrosis or pseudoprogression, can appear identical to tumor in GBM patients [4]. Pseudoprogression is common in the first 12 weeks after chemoradiation, whereas radiation necrosis is comparatively rare and typically manifests after 6 months or following re-irradiation [5]. Throughout this study, both pseudoprogression and radiation necrosis are therefore classed as TRC, whereas tumor-positive disease (TP) denotes residual or recurrent tumor. Distinguishing TP from TRC in clinical practice is a major challenge, as overcalling TRC as TP can lead to therapy discontinuation and unnecessary interventions, while misdiagnosing TP can delay treatment or reoperation and negatively impact patient management [6]. The current method used to assist with the distinction is the Response Assessment in Neuro-Oncology (RANO 2.0) criteria, which primarily rely on changes in contrast enhancement for IDH-wildtype GBM. T2/FLAIR is evaluated separately and is reserved for IDH-mutant non-enhancing gliomas [7]. Because both TP and TRC can show contrast enhancement and surrounding edema from disruption of the blood–brain barrier, conventional MRI often cannot separate them, and qualitative signs such as the soap-bubble appearance of TRC are poorly reproducible [8].

Advanced MRI perfusion imaging has been thoroughly studied as a means of resolving diagnostic uncertainty because it provides additional insights into lesion vascularity and microvascular proliferation [9]. Dynamic susceptibility contrast (DSC) is currently the most widely studied method for assessing treatment response in high-grade gliomas, as it measures cerebral blood volume, an indicator of tumor angiogenesis [10]. Prior research studies have demonstrated its value in differentiating true progression from TRC, although variability in acquisition, leakage correction, and thresholding limits standardization across centers [11]. DSC-derived fractional tumor burden maps have been proposed as a quantified imaging biomarker for viable tumor and have been correlated with the histologic tumor fraction in GBM [12]. Arterial spin labeling (ASL) offers a non-contrast method for measuring cerebral blood flow, avoiding gadolinium use. This makes it useful for repeated monitoring or patients allergic to contrast agents. However, its effectiveness compared with DSC in post-treatment GBM has not yet been fully established [13].

Radiomics provides a quantitative approach for extracting high-dimensional imaging features related to intensity, shape, and spatial texture [14]. In neuro-oncology, radiomics has been applied to tumor classification, molecular prediction, prognosis, and assessment of treatment response [15]. For the TP-versus-TRC question, Sun et al. reported 72.8% accuracy with post-contrast T1-weighted radiomics [16], and McKenney et al. reported 81% accuracy for pseudoprogression with post-contrast T1-weighted and dynamic contrast-enhanced perfusion features, improving to an AUC of 0.89 when MGMT promoter methylation status was added [17]. We previously reported a multiparametric model on the same public cohort that combined post-contrast T1-weighted, FLAIR, diffusion and DSC features [18]. Because these models combined several sequences and, in some cases, molecular data, the contribution of DSC perfusion alone could not be isolated. This matters in practice: many centers lack standardized multiparametric protocols, whereas DSC perfusion is widely used in post-treatment GBM surveillance.

We hypothesized that DSC perfusion radiomics alone can discriminate TP from TRC without additional sequences or clinical variables. The rationale is biological. Contrast enhancement reflects breakdown of the blood–brain barrier, which occurs in both conditions. Perfusion imaging instead reflects the blood supply of the lesion, which differs between them: recurrent tumor forms new vessels and has a higher blood volume, whereas radiation injury damages vessels and lowers blood volume [19]. The study had four aims: first, to measure how well DSC perfusion radiomics performs on its own when training and test patients are kept strictly separate; second, to test whether the results change when a different DSC perfusion map from the same scans is used; third, to compare DSC with ASL, the two clinically available perfusion techniques, in the same patients and lesions; and fourth, to fix the model, a soft-voting ensemble, and the rule used to select it before the test data were examined, so that test performance is not biased by model choice. Feature-importance analysis and a review of individual cases were used to show which perfusion patterns drove the predictions and where the model failed.

## 2. Materials and Methods

### 2.1. Study Design and Cohort

This retrospective study investigated the diagnostic value of DSC perfusion MRI radiomics as a standalone candidate imaging biomarker for differentiating TP from TRC in post-treatment glioblastoma. As an exploratory secondary analysis, ASL radiomics was evaluated as an independent perfusion-based input and directly compared with DSC-only radiomics in patients with both modalities available. Because delayed recognition of TP may postpone treatment escalation, TP was treated as the positive class for all model development and performance reporting.

The initial UCSD-PTGBM dataset included 243 post-treatment MRI examinations from 178 patients [20]. Before model development, 19 TP examinations from 16 patients were excluded to reduce potential confounding. Of these, 9 examinations were excluded because bevacizumab had been administered within 28 days prior to MRI, and 10 examinations were excluded because they involved IDH-mutant GBM [21,22]. Because the dataset included longitudinal imaging, exclusions were applied at the examination level. Therefore, some patients had one MRI examination excluded, but contributed other eligible MRI examinations to the final analysis. After these exclusions, the final analytic DSC cohort included 224 MRI examinations from 168 patients. This analytic cohort has been used previously by our group in a multiparametric analysis of the same clinical question [18]; the present study is independent of it in imaging input, data split, unit of analysis and model class, and no result, model, table or figure is reproduced from it.

All examinations were acquired at two institutions affiliated with UCSD, using 3 T GE scanners (Signa Excite HDx and Discovery MR750, 8-channel head coil; GE Healthcare, Waukesha, WI, USA) with a standardized DSC gradient-echo EPI protocol (TE/TR = 35/1600 ms, flip angle = 90°, 5 mm slice thickness with 1 mm intersection gap, 22 axial slices, FOV = 25 cm, matrix = 96 × 96), following a single gadolinium-based contrast injection protocol (0.1 mmol/kg gadobenate dimeglumine (MultiHance; Bracco Diagnostics, Monroe Township, NJ, USA) or gadobutrol (Gadavist; Bayer HealthCare, Whippany, NJ, USA), 2–3 mL/s power injection). Protocol consistency across sites reduces, but does not eliminate, potential scanner-related variability in radiomic features.

Ground-truth labels were assigned based on UCSD-PTGBM reference annotations, in which post-treatment MRI time points were classified as residual/recurrent tumor or post-treatment change. This classification relied on RANO-based clinical evaluation, longitudinal imaging follow-up, and, when available, histopathologic confirmation. According to the dataset descriptor, 45 of the 192 tumor-positive time points in the source collection were pathology-proven [23]; because the released files do not identify which ones, between 26 and 45 (15.0–26.0%) of the 173 tumor-positive examinations retained here were histopathologically confirmed. Of the 51 TRC examinations, 6 (11.8%) were biopsy-proven pseudoprogression, 9 (17.6%) clinically confirmed pseudoprogression, 7 (13.7%) radiation necrosis, confirmed clinically according to the descriptor, and 29 (56.9%) non-specific post-treatment change established by clinical and imaging follow-up. Overall, histopathology confirmed between 32 (14.3%) and 51 (22.8%) of the 224 examinations; all other labels rest on clinical and imaging follow-up (Supplementary Table S5). In this study, exams labeled as residual or recurrent tumor were grouped as the positive class, while post-treatment change was considered the negative class (TRC), including pseudoprogression, radiation necrosis, and non-specific post-treatment alterations. Although residual tumor, recurrence, and progressive disease are different clinical conditions, they are collectively categorized as tumor-positive within this standard, distinguishing them from treatment-related changes. This grouping addresses the main clinical question, whether an abnormality indicates tumor-positive disease requiring active treatment or a treatment effect that can be observed or in continued therapy. Cohort details are summarized in Table 1.

### 2.2. Patient-Level Data Split

To prevent leakage from repeated longitudinal examinations, all data were split at the patient level. All MRI examinations from the same patient were assigned exclusively to either the training/internal-validation cohort or the independent held-out test cohort, with no patient overlap between cohorts.

The training/internal-validation cohort included 109 patients and 143 MRI examinations. The independent held-out test cohort included 59 patients and 81 MRI examinations. The patient-level and examination-level distribution of TP and TRC cases is shown in Table 2. The size and class balance of the cohort are fixed by the public collection, in which only 51 of 243 time points were TRC [23]; no further TRC cases were available. Approximately one-third of patients were therefore allocated to the test set, so that at least 10 TRC patients were available for an independent test while leaving 19 for training. The consequences of this imbalance for the precision of the estimates are addressed in the Discussion.

All preprocessing, imputation, variance filtering, feature ranking, redundancy reduction, hyperparameter optimization, classifier selection, and threshold selection were performed using only the training/internal-validation data. The held-out test set was used only for the final locked evaluation.

### 2.3. Preprocessing and Radiomic Feature Extraction

Radiomic features were derived from the whole-lesion tumor segmentation masks distributed with the UCSD-PTGBM collection. These BraTS-standard masks were generated by a deep-learning segmentation tool, manually corrected by trained radiologists and reviewed by a board-certified neuroradiologist, with cases returned for correction until the neuroradiologist approved them [23]. We used them unchanged and did not re-segment any lesion, so no additional observer variability was introduced. For feature extraction, all mask labels (enhancing tumor, non-enhancing/necrotic core, surrounding FLAIR hyperintensity and, where present, resection cavity) were merged into a single whole-lesion region. The perfusion input to the primary radiomics pipeline was the leakage-corrected DSC cerebral blood volume map (file suffix CBV_LC) supplied with that collection [20]. These maps were generated by the dataset providers and were used as distributed. As described in the collection’s data descriptor, the raw dynamic susceptibility contrast series were processed with the MATLAB DSC toolbox to compute cerebral blood volume using a leakage-correction algorithm following Boxerman et al., with the arterial input function selected automatically on the middle cerebral artery by recursive cluster analysis, and all processed perfusion data were manually reviewed and reprocessed where necessary for quality [23,24,25]. Leakage correction is important because contrast extravasation through a disrupted blood–brain barrier otherwise artificially lowers rCBV in high-grade glioma, and only leakage-corrected rCBV has been shown to correlate with histopathologic grade [24]. No other DSC-derived perfusion map contributed to the primary rCBV model: the mean transit time map was not used at all, and the cerebral blood flow map derived by singular value decomposition (SVD) deconvolution (file suffix CBF_svd), for which the descriptor does not specify a leakage-correction step, was evaluated separately in a perfusion-map sensitivity analysis described below. The 1223-feature DSC pool was extracted from a single three-dimensional perfusion volume per examination. DSC and ASL perfusion images were resampled to align with the segmentation mask grid at 1 mm isotropic resolution, using B-spline interpolation for images and nearest-neighbor interpolation for masks. N4 bias-field correction, which is designed for structural images, was not applied to the perfusion maps. Images were normalized by within-mask z-scoring, followed by the PyRadiomics intensity normalization (scale 100). Features were extracted with a fixed bin width of 25 from the original images and from Laplacian-of-Gaussian (LoG; sigma 0.5, 1.0, 2.0 and 3.0 mm) and wavelet-filtered images. The primary analysis used features from the DSC rCBV maps, while an exploratory comparison involved extracting features from the corresponding ASL perfusion images.

A fixed bin width of 25 was selected in accordance with Image Biomarker Standardization Initiative (IBSI)-recommended discretization practices for MRI-derived quantitative maps, balancing histogram resolution against noise sensitivity [26]. A fixed bin width, rather than a fixed number of bins, keeps the meaning of each gray level constant across patients, so that texture features compare like with like. Within-mask z-score normalization was applied to standardize intensity scale differences across patients prior to feature extraction, given the non-quantitative signal units of DSC perfusion images. Because the normalization statistics are computed inside the mask, a different segmentation also changes the normalized intensities; this combined effect was quantified in the segmentation-perturbation analysis (Section 2.8). The Spearman correlation threshold and the number of top-ranked features were selected empirically within each classifier pipeline during hyperparameter optimization, to balance redundancy reduction against retained feature information; they were treated as tunable pipeline parameters rather than fixed a priori. The optimized values were |ρ| < 0.70 for HistGradientBoosting, |ρ| < 0.85 for elastic-net logistic regression and RBF-SVM, and |ρ| < 0.75 for Random Forest, with 0.85 for Extra Trees.

Feature extraction was performed with PyRadiomics. Features were extracted from original images and from derived images generated using Laplacian-of-Gaussian (LoG) and wavelet filters. The extracted feature set included first-order intensity statistics, shape descriptors, and texture features from gray-level co-occurrence matrix (GLCM), gray-level run-length matrix (GLRLM), gray-level size-zone matrix (GLSZM), gray-level dependence matrix (GLDM), and neighboring gray-tone difference matrix (NGTDM). The initial DSC-only feature pool contained 1223 radiomic features.

### 2.4. Feature Processing and Redundancy Reduction

Feature processing was performed separately within each classifier pipeline using training data only. Missing values were imputed using the median value from the training set. Features with near-zero variance were removed. The remaining features were ranked using a univariate ANOVA F-test, and the number of top-ranked features retained (top-K) was treated as a tunable pipeline parameter rather than fixed in advance.

**Figure 1:**
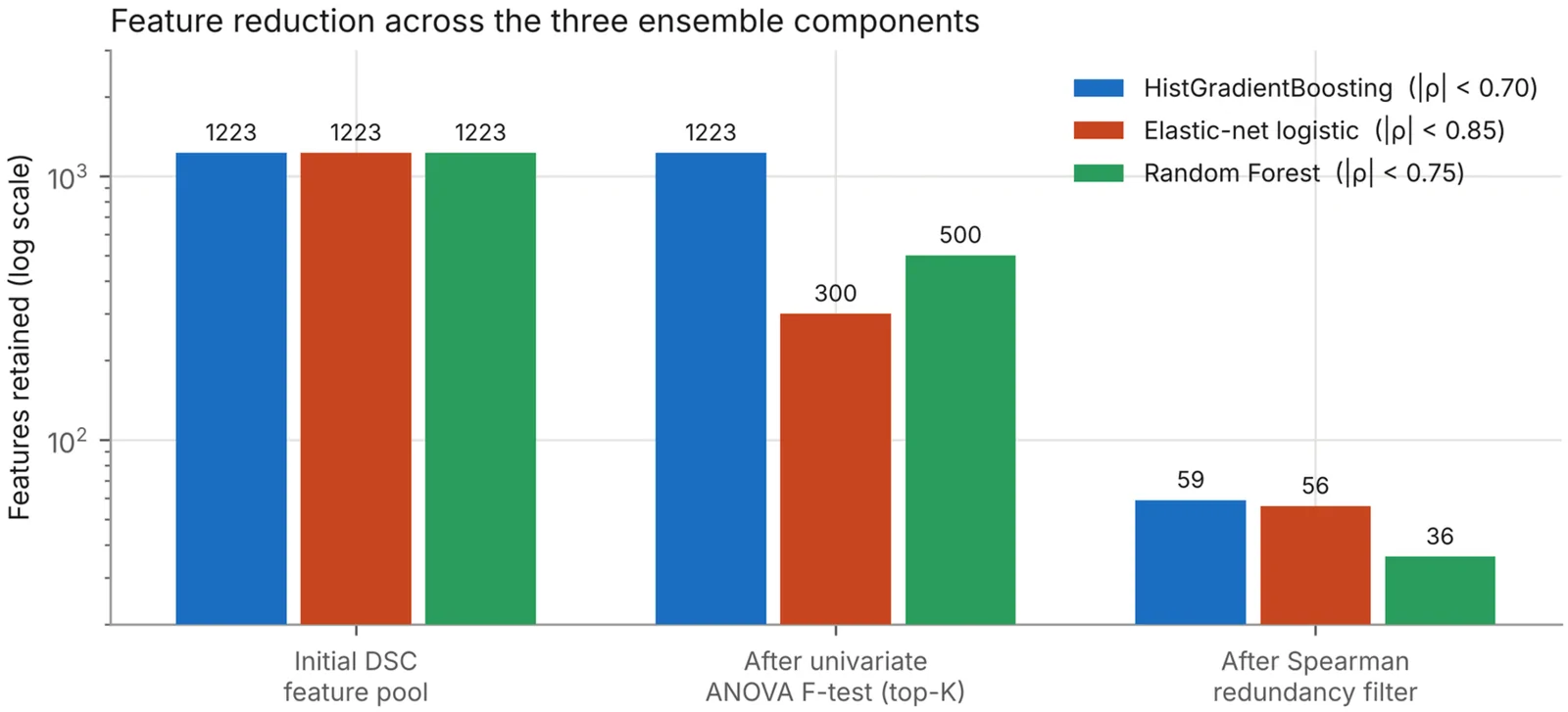
Feature reduction across the three ensemble components. The initial DSC rCBV feature pool of 1223 features was reduced to 59 for HistGradientBoosting, 56 for elastic-net logistic regression, and 36 for Random Forest, using classifier-specific univariate top-K selection followed by Spearman redundancy filtering at the cut-off shown for each component.

To reduce collinearity, a Spearman correlation filter was then applied to the top-ranked features. For pairs of features exceeding the pipeline’s absolute Spearman correlation threshold, the lower-ranked feature was removed and the higher-ranked feature retained. Both the top-K count and the correlation threshold were optimized within each classifier pipeline on training data only, so the final feature sets differ across pipelines. For the three classifiers comprising the final ensemble, the HistGradientBoosting pipeline retained 59 features (top-K 1223; |ρ| < 0.70), the elastic-net logistic regression pipeline retained 56 features (top-K 300; |ρ| < 0.85), and the Random Forest pipeline retained 36 features (top-K 500; |ρ| < 0.75) (Figure 1). The comparator pipelines retained 56 features for RBF-SVM (top-K 300; |ρ| < 0.85) and 163 features for Extra Trees (top-K 1223; |ρ| < 0.85).

### 2.5. Model Development and Selection

The primary model was a soft-voting ensemble, i.e., the average of the predicted probabilities of several classifiers. An ensemble was chosen over a single classifier for three reasons, all fixed before the test data were examined. First, the candidate classifiers learn in different ways (gradient-boosted trees, bagged trees, a penalized linear model and a kernel method), and averaging them reduces the variance of any one model, which matters with a small, imbalanced training set (109 patients, 19 with TRC). Second, picking the single best classifier on a small internal-validation set risks choosing on noise; averaging over a predefined set of members is less sensitive to this. Third, an ensemble defined by a rule applied only to internal cross-validation predictions can be locked before testing. The purpose of the ensemble was therefore a stable, prespecified model, not the highest possible test AUC, and it was not expected to outperform every individual classifier. The candidate ensembles (unweighted and AUC-weighted averages of all five classifiers, of the top two and top three classifiers ranked by internal AUC or by internal precision–recall AUC, and of predefined tree-based and nonlinear subsets) and the individual classifiers are listed in Supplementary Table S3; the ensemble with the highest internal patient-level AUC was selected. For the primary rCBV analysis, this was the unweighted average of a HistGradientBoosting classifier, an elastic-net logistic regression and a Random Forest; for the secondary CBF analysis, it was the unweighted average of a radial-basis-function support vector machine (RBF-SVM), an Extra Trees classifier and a Random Forest. Hyperparameters and pipeline settings were optimized through 200 Optuna trials per classifier. Internal validation employed a 5-fold StratifiedGroupKFold cross-validation, grouped by patient ID. To address class imbalance, balanced sample weights were used during training, inversely proportional to class frequency (training TP:TRC ratio approximately 4.7:1; 90 vs. 19 patients). These weights were computed within each fold to prevent leakage. While this adjustment corrects the loss function’s class contribution, it does not increase the number of independent TRC observations for training. Scan-level probabilities were collapsed to the patient level by taking the maximum probability across a patient’s examinations before ensemble averaging. Two operating points were defined on out-of-fold predictions only: a primary threshold maximizing the Youden index, and a higher-sensitivity threshold, defined as the most sensitive out-of-fold threshold maintaining out-of-fold specificity of at least 0.70. Platt scaling was fitted on training out-of-fold predictions only and evaluated as a calibration sensitivity analysis; because it produced only a marginal improvement in Brier score, the uncalibrated ensemble probabilities were retained for the primary diagnostic analysis, and no calibration step was fitted on held-out data. Each held-out evaluation was performed once, after all of the above was fixed.

### 2.6. Model Evaluation and Modality Comparison

Final diagnostic performance was assessed on an independent, held-out test cohort. For patients with multiple follow-up exams, data were aggregated at the patient level before calculating metrics. A patient was considered a TP if at least one exam confirmed TP. The predicted probability at the patient level was defined as the highest TP probability among that patient’s exams. The Youden threshold, derived from training data, was then applied to these aggregated probabilities. The primary endpoint was the patient-level held-out area under the receiver operating characteristic curve (AUC) of the leakage-corrected rCBV ensemble. Secondary metrics included sensitivity for TP, specificity for TRC, balanced accuracy, F1 score, Matthews correlation coefficient (MCC), precision–recall AUC (PR-AUC), positive predictive value (PPV) and negative predictive value (NPV). Confidence intervals for AUC, PR-AUC, balanced accuracy, F1, MCC and the Brier score were estimated with 2000 bootstrap resamples at the patient level; for sensitivity, specificity, PPV and NPV, exact Clopper–Pearson binomial intervals are reported instead, because these are simple proportions with small denominators (10 TRC patients) for which bootstrap intervals are degenerate. Two further comparisons were defined before held-out evaluation. First, a perfusion-map sensitivity analysis compared the primary rCBV ensemble with the corresponding CBF ensemble across the identical 59 held-out patients using a paired DeLong test, to quantify the influence of the perfusion map and its leakage-correction status. Second, for the exploratory comparison of perfusion modalities, DSC and ASL performance was evaluated on the shared ASL-available held-out subset using a paired DeLong test; because the ASL parameter available in this collection is cerebral blood flow, the primary modality comparison was made modality-matched, contrasting the DSC CBF ensemble with ASL CBF. The contrast between the primary rCBV ensemble and ASL CBF is reported alongside it, but is not parameter-matched and is interpreted accordingly.

### 2.7. Explainability Analysis

Since the primary model was a heterogeneous ensemble, we used model-agnostic permutation feature importance for interpretability. Each feature was randomly permuted five times in the held-out cohort, and importance was measured by the mean reduction in patient-level AUC. This method identified the DSC rCBV radiomic features most strongly associated with model performance, avoiding reliance on classifier-specific importance metrics. To complement this global, statistical view with a case-level assessment, we additionally reviewed representative held-out patients qualitatively against their conventional imaging. One patient was selected from each cell of the held-out confusion matrix using a rule fixed before inspection: the patient with the median predicted probability within that cell. Because the primary Youden operating point produced no false positives, confusion-matrix cells for this figure were defined at the higher-sensitivity operating point. For each selected patient, post-contrast T1, FLAIR, the DSC rCBV map that fed the radiomics pipeline, and the ASL CBF map where acquired are displayed at the slice of maximal tumor cross-section with the segmentation contour overlaid, annotated with the predicted probability, the decision at the applied threshold, the reference-standard basis, and the segmented lesion volume. This review was performed by the study team for face validity and was not a formal reader study; no independent expert readers were recruited and no reader performance was measured.

### 2.8. Robustness and Overfitting Assessment

Three additional analyses, performed on the locked pipeline without changing the model, assessed robustness. First, to approximate segmentation variability, each whole-lesion mask was eroded and dilated by 1 mm and by 2 mm with a spherical kernel, features were re-extracted from the rCBV map with identical settings (including within-mask normalization), and agreement across the five mask conditions was quantified for every feature with a two-way mixed-effect, consistency, single-measure intraclass correlation coefficient, ICC(3,1), interpreted as poor (<0.50), moderate (0.50–0.75), good (0.75–0.90) or excellent (>0.90) [27]. Second, to assess the stability of feature selection, the selection steps of each ensemble component (median imputation, variance filtering, ANOVA top-K ranking and Spearman redundancy filtering, with the locked top-K and correlation settings) were repeated within the training portion of each of the five internal cross-validation folds; agreement between folds was summarized as the pairwise Jaccard index and as the number of folds in which each feature was selected, with the Jaccard index expected for random feature sets of the same size as a chance reference. Third, as an overfitting control, the training labels were randomly permuted 200 times and the complete cross-validated pipeline (feature selection and classifier fitting, with hyperparameters fixed at their optimized values) was re-run each time to obtain a null distribution of the ensemble’s internal patient-level AUC.

### 2.9. Software and Reproducibility

All analyses were performed in Python 3.12.1. Radiomic feature extraction was performed using PyRadiomics 3.1.1, and machine-learning pipelines were implemented using scikit-learn 1.8.0. Hyperparameter optimization was performed with Optuna 4.8.0. Data processing and statistical analyses used NumPy 2.4.4, pandas 3.0.2, and SciPy 1.17.1. The random seed was fixed at 42 throughout. Analysis code and the locked patient-level split are available from the corresponding author on reasonable request.

## 3. Results

### 3.1. Model Configuration and Feature Reduction

The final DSC-only soft-voting ensemble integrated three optimized classifier pipelines: HistGradientBoosting, elastic-net logistic regression, and Random Forest. During feature reduction, the original set of 1223 DSC rCBV features was narrowed to component-specific subsets: 59 features for HistGradientBoosting (top-K 1223, |ρ| < 0.70), 56 for elastic-net logistic regression (top-K 300, |ρ| < 0.85), and 36 for Random Forest (top-K 500, |ρ| < 0.75) (Figure 1). Platt scaling produced only a marginal improvement in calibration; the Brier score of the ensemble shifted from 0.135 to 0.129, with AUC unchanged. Consequently, raw ensemble probabilities were used for the main performance assessment and secondary metrics.

### 3.2. Held-Out Patient-Level Performance

The DSC-only ensemble showed moderate to good diagnostic ability on the patient-level test set. During internal validation, it achieved an out-of-fold AUC of 0.832 at the patient level. On the independent test set, the model achieved a patient-level AUC of 0.790, with a 95% bootstrap confidence interval of 0.667 to 0.891. The confidence interval remains wide, which is attributable to the limited sample size of TRC (*n* = 10 patients), where a single misclassification shifts specificity by 10 percentage points.

**Figure 2:**
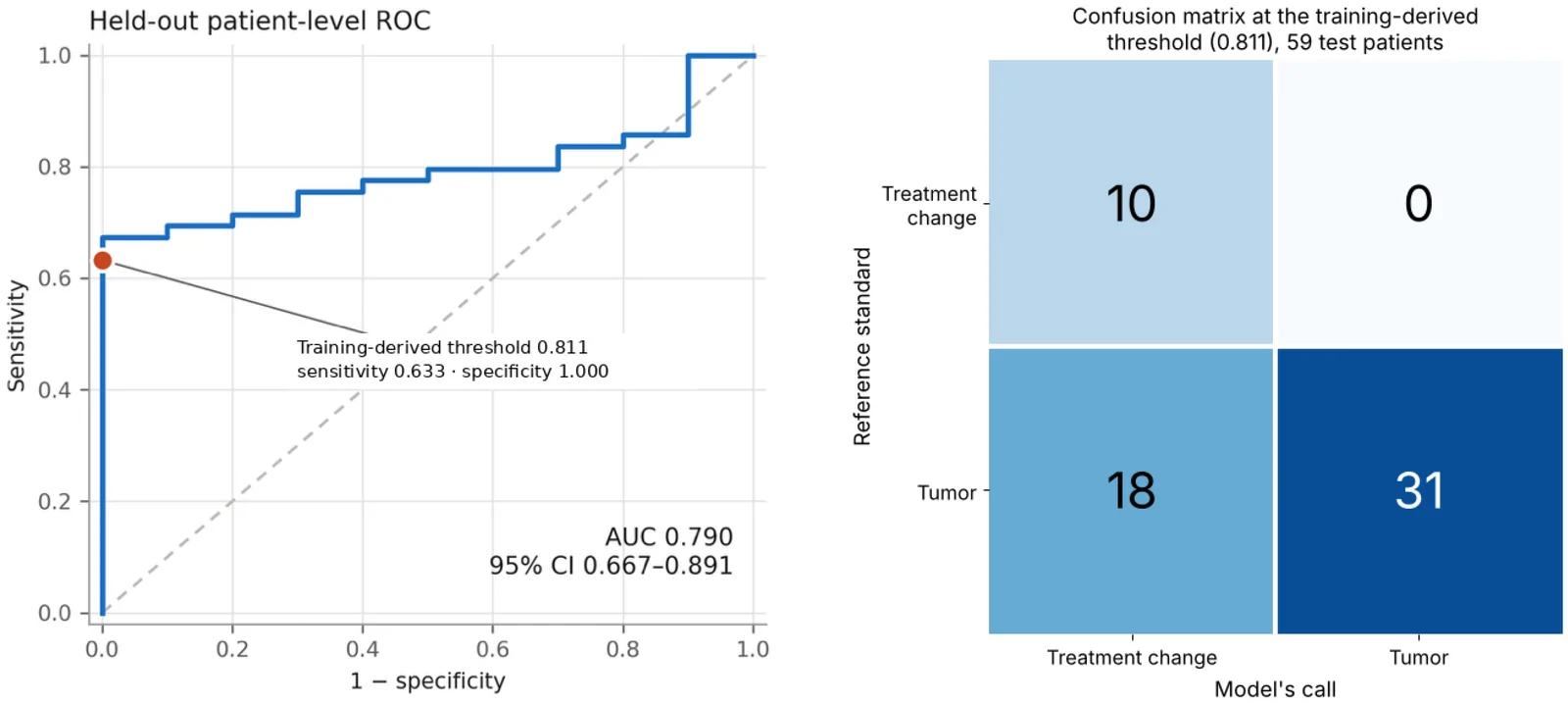
Patient-level performance of the locked DSC rCBV soft-voting ensemble on the independent held-out test set. The ROC curve shows a held-out AUC of 0.790 (95% CI 0.667–0.891). The confusion matrix shows classifications generated using the training-derived Youden threshold (0.811), with 31 true positives, 10 true negatives, 0 false positives and 18 false negatives at the patient level. Rows give the reference standard and columns the model’s call; “Tumor” denotes tumor-positive disease and “Treatment change” denotes treatment-related change.

At the training-derived Youden threshold (0.811), the model correctly identified 31 of 49 patients with tumor (sensitivity 63.3%, 95% CI 48.3–76.6%) and all 10 patients with TRC (specificity 100%, 95% CI 69.2–100%). Every positive call was correct (PPV 100%, 95% CI 88.8–100%), but only 10 of 28 negative calls were correct (NPV 35.7%). Balanced accuracy was 81.6%, F1 score 77.5%, MCC 0.475 and PR-AUC 0.958. At the predefined higher-sensitivity threshold (0.727), sensitivity rose to 69.4% and specificity fell to 80.0% (34 true positives, 8 true negatives, 2 false positives and 15 false negatives; PPV 94.4%, NPV 34.8%, balanced accuracy 74.7%, MCC 0.380) (Table 3, Figure 2). Operating characteristics across a range of training-derived thresholds are provided in Supplementary Table S2.

Relative to the DSC-only single-classifier baselines, the soft-voting ensemble did not achieve the highest held-out AUC, but it was selected on internal out-of-fold performance, as defined before held-out evaluation, and it produced the highest specificity and PPV of any model at its primary operating point (Table 3, Figure 2).

### 3.3. Classifier Architecture Comparison

The performance of individual classifiers varied across architectures (Table 4). On internal cross-validation, HistGradientBoosting ranked highest (AUC 0.861), followed by elastic-net logistic regression (0.833), Extra Trees (0.822), Random Forest (0.810) and RBF-SVM (0.806). On the test set, this order did not hold: Random Forest had the highest AUC (0.839), and HistGradientBoosting, first internally, had the lowest (0.737). The ensemble did not differ significantly from any individual classifier (paired DeLong tests, all *p* > 0.20; Supplementary Table S1), including Random Forest (ΔAUC −0.049, 95% CI −0.125 to 0.027, *p* = 0.207). Because the test set was reserved for a single evaluation, it was not used to choose among models, and the instability of the individual rankings between internal and test data supports that choice. We therefore report that the ensemble showed no statistically significant difference from the best individual classifier; this non-significant result does not establish equivalence, and the ensemble was not better than that classifier.

### 3.4. Permutation-Based Feature Importance

**Figure 3:**
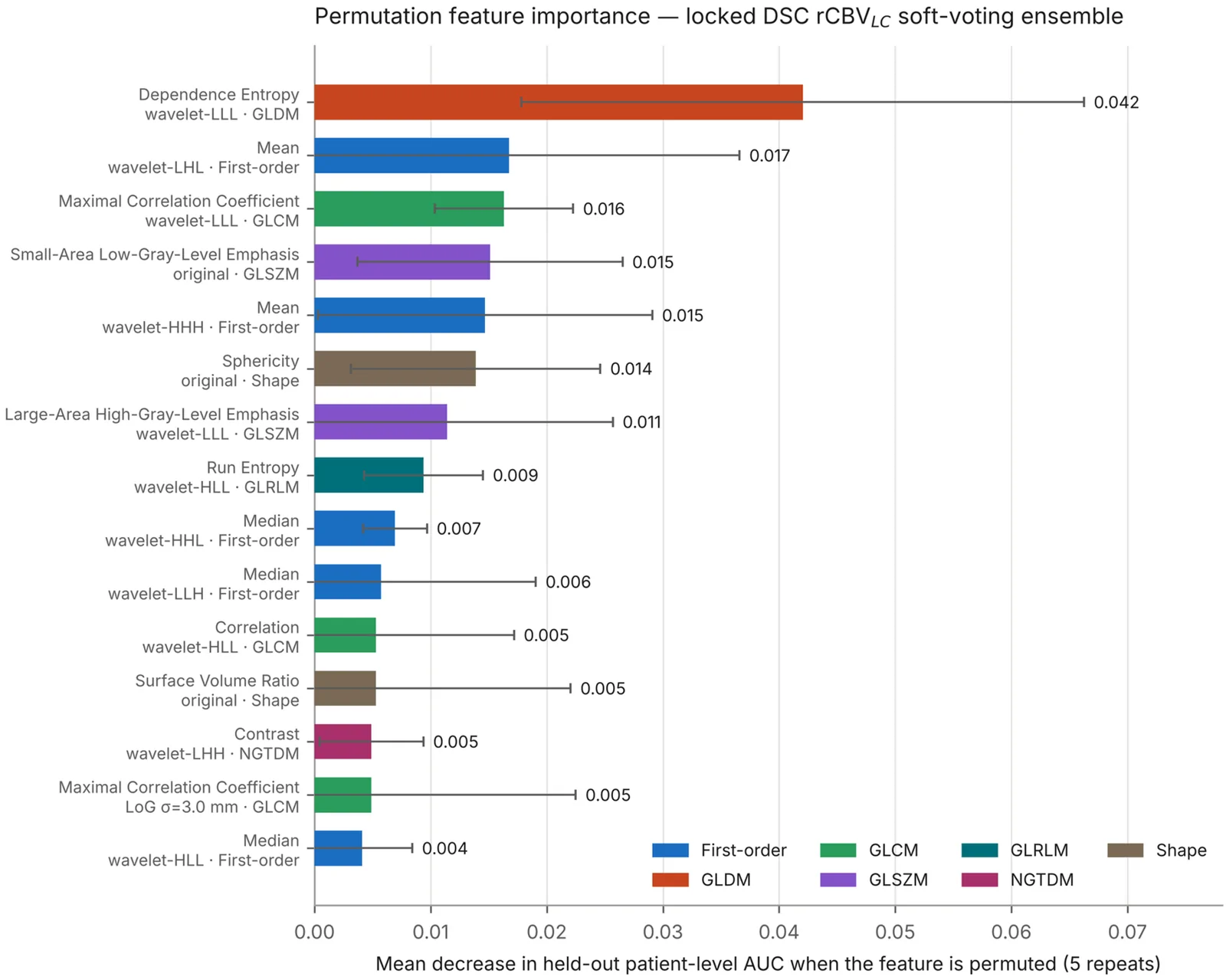
Permutation feature importance for the locked DSC rCBV soft-voting ensemble. Features are ranked by the mean decrease in patient-level AUC after random permutation across five repeats. Horizontal error bars indicate the standard deviation across repeats. Bars are colored by radiomic feature family.

Permutation feature importance indicated that the model’s predictions were linked to multiscale DSC rCBV heterogeneity, with contributions from first-order intensity, texture and lesion-shape descriptors (Figure 3, Table 5). The single most influential feature was wavelet-filtered GLDM dependence entropy (mean AUC decrease 0.042), followed by wavelet-filtered first-order mean, wavelet-filtered GLCM maximal correlation coefficient, GLSZM small-area low-gray-level emphasis on the original image, and a second wavelet-filtered first-order mean. Of the 15 most influential features, five were first-order, three GLCM, two GLSZM, two shape, and one each GLRLM, GLDM and NGTDM. The appearance of original-image shape descriptors (sphericity, surface-to-volume ratio) among the top features indicates that lesion geometry, and not perfusion intensity and texture alone, contributed to discrimination. Individual importances were small in absolute terms and their resampling standard deviations overlapped substantially, so this ranking should be read as identifying broad feature classes rather than individually indispensable features.

### 3.5. Perfusion-Map Sensitivity Analysis

**Figure 4:**
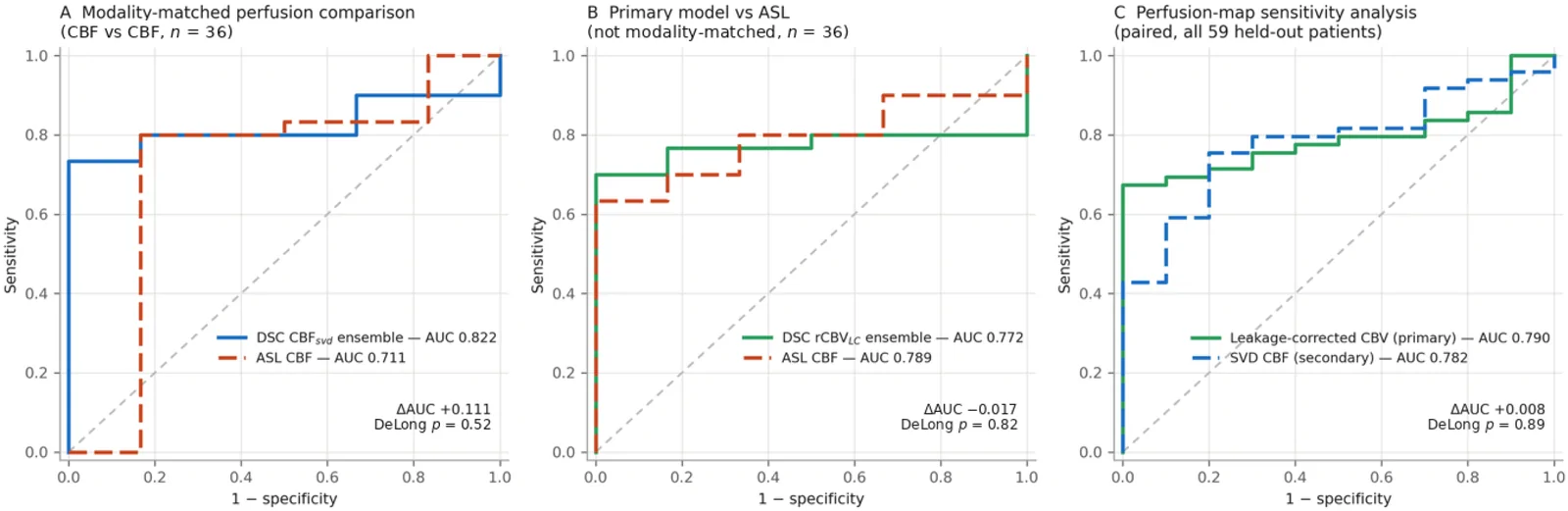
Perfusion comparisons on the held-out cohort. (**A**) Modality-matched exploratory comparison in the 36 patients with available ASL: the DSC CBF ensemble achieved an AUC of 0.822 and the ASL-only model 0.711 (delta AUC +0.111, paired DeLong *p* = 0.52). (**B**) The primary leakage-corrected rCBV ensemble contrasted with ASL CBF in the same subset (0.772 vs. 0.789; delta AUC −0.017, *p* = 0.82); this contrast is not parameter-matched. (**C**) Perfusion-map sensitivity analysis across all 59 held-out patients: the leakage-corrected rCBV ensemble (AUC 0.790) and the CBF ensemble (AUC 0.782) did not differ significantly (paired delta AUC +0.008, DeLong *p* = 0.89), despite differing markedly in operating characteristics and in selected classifier membership.

Because the choice of perfusion map and its leakage correction is a known source of between-study differences for this task, we repeated the entire locked pipeline on the CBF map from the same examinations, patients and split (Table S4, Figure 4C). The CBF ensemble, selected by the identical predefined rule, comprised an RBF-SVM, an Extra Trees classifier and a Random Forest classifier, and achieved an out-of-fold AUC of 0.820 and a held-out AUC of 0.782 (95% CI 0.632–0.900). The AUC did not differ significantly between the two maps in the same 59 held-out patients (paired ΔAUC +0.008, 95% CI −0.107 to +0.124, DeLong *p* = 0.890); this does not establish equivalence. The operating characteristics, however, differed: at each model’s own training-derived Youden threshold, the rCBV ensemble gave 63.3% sensitivity with 100% specificity (31/10/0/18) whereas the CBF ensemble gave 75.5% sensitivity with 80.0% specificity (37/8/2/12), and the selection rule defined before held-out evaluation chose different classifier members for each map. In practice, the AUCs of the two maps did not differ significantly, but the decision threshold and the selected model cannot be transferred from one map to the other.

### 3.6. Exploratory DSC-Only Versus ASL-Only Comparison

The exploratory modality comparison was conducted on a shared subset of 36 patients with available ASL imaging, comprising 30 with TP and 6 with TRC. In the modality-matched comparison, in which both models used cerebral blood flow, the DSC CBF ensemble achieved a patient-level AUC of 0.822 and the ASL-only radiomics model an AUC of 0.711 (delta AUC +0.111, paired DeLong *p* = 0.524), numerically favoring DSC. When the primary leakage-corrected rCBV ensemble was contrasted with ASL CBF instead, the ordering reversed (0.772 vs. 0.789; delta AUC −0.017, *p* = 0.819), but this contrast is not parameter-matched and the two perfusion quantities are not physiologically equivalent. Neither comparison approached statistical significance. Given the small sample size, the marked class imbalance (6 TRC patients) and the wide confidence intervals, these comparisons should be regarded as exploratory and as providing no evidence that either perfusion modality outperforms the other for this task (Figure 4, Table 6).

### 3.7. Qualitative Case Review

**Figure 5:**
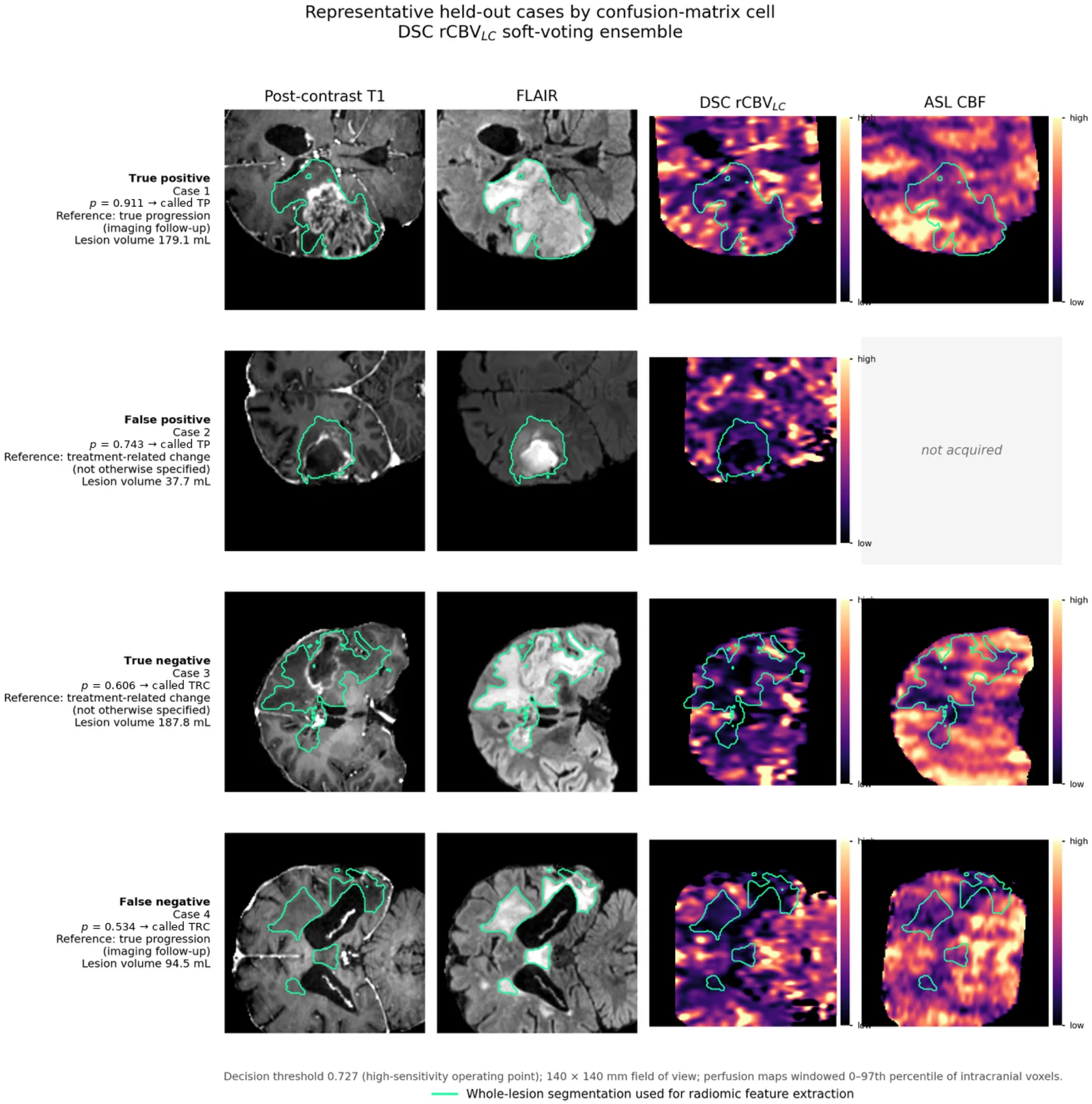
Representative held-out cases by confusion-matrix cell for the DSC rCBV soft-voting ensemble. One patient per cell was selected using a rule fixed before inspection: the patient with the median predicted probability within that cell. Because the primary Youden threshold produced no false positives, cells are defined at the predefined higher-sensitivity operating point (threshold 0.727). Each row shows post-contrast T1, FLAIR, the leakage-corrected DSC rCBV map that fed the radiomics pipeline, and the ASL CBF map where acquired, at the slice of maximal tumor cross-section, with the whole-lesion segmentation contour overlaid in green. Row annotations give the case label, the ensemble predicted probability and resulting call, the reference-standard basis, and the segmented lesion volume. Perfusion maps are windowed to the 0–97th percentile of intracranial voxels; field of view 140 × 140 mm.

Case-level review of representative held-out patients is shown in Figure 5. The true-positive exemplar (Case 1, predicted probability 0.911) was a large 179.1 mL lesion with markedly heterogeneous intralesional rCBV. The false-positive exemplar (Case 2, 0.743, 37.7 mL) showed thick irregular peripheral enhancement with a central rCBV-poor cavity and hyperperfused rim, an appearance that may be difficult to distinguish from recurrent tumor even on expert review, although no formal reader assessment was performed. The true-negative exemplar (Case 3, 0.606, 187.8 mL) was large but comparatively homogeneous and low in rCBV. The false-negative exemplar (Case 4, 0.534, 94.5 mL) is the most instructive: it was not a small, subtle recurrence but a sizeable periventricular lesion with visible enhancement, indicating that the model’s misses are not confined to borderline cases. Segmentation contours followed the enhancing and FLAIR-hyperintense abnormality on the conventional sequences in all four cases, with no obvious segmentation or registration artifact on visual inspection. Among held-out TRC patients, the lowest predicted probabilities were assigned to the cases with a named confirmation route (clinically confirmed radiation necrosis, 0.498; biopsy-proven pseudoprogression, 0.665), while the highest were assigned to cases whose reference standard was not further specified.

### 3.8. Robustness and Overfitting Analyses

Within the training cohort (109 patients, 143 examinations), feature selection was reproducible across the five internal cross-validation folds (Supplementary Table S6). For HistGradientBoosting, the mean pairwise Jaccard overlap between folds was 0.81 (range 0.74–0.87), and 98.3% of its 59 final features were selected in at least three of five folds. For elastic-net logistic regression and Random Forest, whose top-K step retained fewer candidate features, overlap was 0.40 and 0.41, against approximately 0.02 expected by chance, and 75.0% and 80.6% of final features recurred in at least three folds. The share of each feature family changed by only a few percentage points between folds, and 13 of the 15 most influential features (Table 5) were selected in all five folds by at least one ensemble component (Supplementary Table S7). In the label-permutation control, the ensemble’s internal AUC with randomly permuted labels had a mean of 0.53 (95th percentile 0.69; maximum 0.80) over 200 permutations, compared with 0.82 for the same fixed pipeline with the true labels (permutation *p* = 0.005), showing that the pipeline did not generate apparent discrimination from noise (Supplementary Table S9); for the locked ensemble, the drop from internal to test AUC was 0.042 (0.832 to 0.790). Under segmentation perturbation (masks eroded or dilated by 1 mm and 2 mm, changing lesion volume by a median of −29% to +60%), the median ICC(3,1) across all 1223 rCBV features was 0.90, and 75.9% of features had good or excellent reliability (ICC ≥ 0.75); restricted to ±1 mm, these values were 0.95 and 84.4%. The 108 features used by the ensemble were less robust (median ICC 0.78, 54.6% ≥ 0.75; ±1 mm: 0.87 and 72.2%). The most influential feature, wavelet dependence entropy, was highly robust (ICC 0.93), as were sphericity (ICC 0.93) and four of the other seven texture features among the top 15, whereas first-order mean and median features from high-frequency wavelet bands were sensitive to the boundary (ICC −0.05 to 0.70), consistent with their values lying close to zero. Overall, 6 of the 15 most influential features had an ICC ≥ 0.75 across all perturbations, and 11 of 15 across ±1 mm (Supplementary Table S8).

## 4. Discussion

Radiomics of leakage-corrected DSC rCBV alone distinguished TP from TRC with a test AUC of 0.790. At the primary threshold, the model raised no false alarms among 10 TRC patients but missed 18 of 49 patients with tumor-positive disease; at the higher-sensitivity threshold, sensitivity rose to 69.4% and specificity fell to 80.0%. In clinical terms, the model behaves as a rule-in rather than a rule-out test. With an NPV of 35.7%, about two of every three patients called “treatment-related change” actually had tumor, so a negative result should prompt continued close follow-up, further imaging or tissue sampling rather than reassurance. Predictive values also depend on prevalence: tumor was present in 83% of test patients, more than in many unselected follow-up populations, in which the NPV would be higher and the PPV lower. The most realistic use is therefore to add confidence to a suspected recurrence when the model is positive, not to exclude recurrence when it is negative.

The features most strongly associated with model performance spanned first-order intensity, texture and shape descriptors, indicating that the model relied on the distribution and spatial organization of rCBV within the lesion rather than on any single global perfusion summary. These patterns are consistent with, though not direct evidence for, the known heterogeneity of recurrent GBM, in which viable tumor, necrosis, edema, hypoxia and abnormal angiogenesis may coexist within the enhancing lesion [12]. This interpretation is supported by prior DSC work showing that leakage-corrected rCBV is higher in recurrent GBM than in radiation necrosis and correlates with histopathologic tumor grade, whereas uncorrected rCBV does not [24,28]. The leading features have plausible biological counterparts. Dependence entropy, the most influential feature, measures how irregular the spatial arrangement of rCBV values is; high values indicate a patchwork of high- and low-blood-volume regions, as expected when proliferating, highly vascular tumor lies next to necrosis and edema. Image-guided biopsies of glioblastoma show that regions with higher rCBV also have higher tumor content, cellular density and proliferation [29], and in treated glioblastoma, rCBV tracks the histologic fraction of recurrent tumor relative to treatment effect [9]. The wavelet-filtered mean and median rCBV features capture blood volume at different spatial scales, the property most directly linked to tumor neoangiogenesis [19]. Small-area low-gray-level emphasis and large-area high-gray-level emphasis describe, respectively, scattered small foci of low blood volume and confluent regions of high blood volume. In radiation necrosis, direct damage to endothelial cells leads to vascular hyalinization, ischemia and necrosis [30], which would be expected to produce fragmented, low-rCBV regions, whereas recurrent tumor tends to form larger, confluent high-rCBV regions. The presence of lesion-shape descriptors among the most influential features is a caution as much as a finding: geometry is not a perfusion property, and its contribution means part of the discrimination may derive from lesion morphology captured by the segmentation rather than from perfusion physiology. Because no radiologic–pathologic correlation was available in this cohort, these feature-to-biology links remain hypotheses.

Multiparametric studies report higher AUCs: 0.90 for post-contrast T1-weighted, FLAIR, diffusion and CBV images combined, although the CBV-only submodel reached 0.80, close to our result [31]; 0.83 for post-contrast T1-weighted imaging combined with clinical data [32]; and 0.905 for diffusion tensor imaging combined with DSC [33]. The cost of relying on DSC alone is therefore roughly 0.04–0.12 AUC, which may be acceptable for centers without standardized multiparametric protocols or reliable capture of clinical variables.

The DSC-versus-ASL comparison is exploratory and should not be used to rank the two techniques. It included only 36 patients, 6 of them with TRC, which gives little statistical power to detect even a moderate difference in AUC; neither comparison was significant, and the direction of the difference reversed when the rCBV model replaced the CBF model. This reversal shows that DSC-versus-ASL comparisons depend on which DSC parameter is used, and that comparing an rCBV-based DSC model with an ASL CBF model is not a like-for-like comparison. Both perfusion techniques have distinct advantages and limitations. ASL is attractive because it does not require gadolinium contrast, provides noninvasive cerebral blood flow estimation, and avoids the contrast-leakage effects that necessitate correction in DSC [34]. However, ASL generally exhibits a lower signal-to-noise ratio than DSC and is more affected by arterial transit time, labeling efficiency, physiological flow variability and patient-specific hemodynamics [10]. Adequately powered, parameter-matched comparisons are needed before any claim of superiority in either direction.

Our findings support DSC radiomics as a candidate quantitative perfusion biomarker, not as an established clinical tool. DSC perfusion is already widely used in neuro-oncology, and DSC-derived fractional tumor burden correlates with the histologic tumor fraction in treated glioblastoma [12]; our model extends this by using the texture and distribution of rCBV across the whole lesion. Its test AUC (0.790), however, lies within the range reported for single DSC metrics such as rCBV thresholds, whose performance varies with acquisition and leakage correction [11], so any advantage of radiomics is more likely to lie in automated, reproducible measurement than in a large gain in accuracy. Whether the model improves on current practice is unproven: it has not been compared with neuroradiologist reading or RANO assessment, has not been tested at other centers or with other scanners and post-processing software, and has not been evaluated prospectively. If validated, its most realistic role would be as a second reader in equivocal cases, alongside radiologist interpretation and RANO assessment. Before clinical use, it requires external multicenter validation, standardized acquisition and post-processing protocols, and a prospective reader study comparing radiologists with and without the model.

This study has several strengths. It isolates DSC perfusion, giving a direct test of what a single, widely available sequence can achieve; it locks the model and its selection rule before testing; it repeats the full analysis on a second perfusion map; and it combines global feature importance with case-level review and with fold-stability, label-permutation and segmentation-perturbation analyses.

This study has limitations. First, it is retrospective, and all data came from one public collection acquired at two affiliated institutions on two GE 3 T scanner models with a single DSC protocol; the model has not been externally validated. Future validation should span other vendors, field strengths, acquisition protocols and post-processing pipelines, apply harmonization where needed, and report results by site and by leakage-correction method. Second, the biomarker is tied to a specific perfusion map produced by the dataset providers (leakage correction following Boxerman et al. and an automatically selected arterial input function) [23,24,25]. Repeating the analysis on the CBF map gave an AUC that did not differ significantly but changed the operating point and the selected classifiers, so a center using a different leakage-correction method, arterial input function or preload strategy could obtain a different operating point, the main source of between-study differences identified in a previous meta-analysis [11]; the preload strategy could not be verified from the descriptor. Third, the cohort is small and imbalanced. The training set contained 19 TRC patients and the test set 10, reflecting the low proportion of TRC in the source collection. Class weighting balances the training loss but cannot create new TRC examples. With 10 TRC test patients, one misclassification changes specificity by 10 percentage points, so the 100% specificity (exact 95% CI 69.2–100%) and the NPV are imprecise and should not be read as stable estimates; we therefore also report balanced accuracy, MCC and PR-AUC, which depend less on prevalence. Fourth, the initial feature pool (1223 features) was large relative to the number of patients. We limited overfitting by performing all feature selection within training folds and locking the model before testing, and the fold-stability and label-permutation analyses did not indicate instability or fitting to noise; the modest decrease from internal to test AUC (0.832 to 0.790) is consistent with limited optimism, but only external data can provide an unbiased estimate. Fifth, sensitivity at the primary threshold was limited (63.3%), which restricts the clinical value of a negative result. Sixth, reference labels rest mainly on clinical and imaging follow-up: histopathology confirmed at most 22.8% of examinations, and more than half of the TRC examinations were non-specific post-treatment change. Seventh, we used the provider segmentations, which were approved through a single consensus process, so inter- and intra-observer variability could not be measured directly; the perturbation analysis simulates boundary variability but not disagreement about which tissue belongs to the lesion. The perturbation analysis also showed that first-order features from high-frequency wavelet bands, several of which ranked among the most influential, were sensitive to boundary changes, and the whole-lesion region included the resection cavity where present. Eighth, the ASL comparison involved only 36 patients, 6 with TRC, and is underpowered. Finally, permutation importance indicates which features the model relies on, not their biological meaning; no radiologic–pathologic correlation was available to confirm the proposed biological links, and patient-level attribution methods remain a target for future work.

## 5. Conclusions

DSC perfusion radiomics alone distinguished tumor-positive disease from treatment-related change in post-treatment glioblastoma with moderate-to-good accuracy (AUC 0.79 in 59 held-out patients). The model was highly specific but missed about one-third of tumors, so a positive result may support a diagnosis of tumor, whereas a negative result cannot exclude it. The AUC did not differ significantly between two different DSC perfusion maps, but the best decision threshold and the selected model differed, so studies should report how perfusion maps were produced. These results are preliminary because the test set was small, most labels relied on follow-up rather than histopathology, and the model has not been validated externally. Multicenter external validation, standardized imaging and post-processing protocols, and prospective comparison with expert neuroradiologists are needed before clinical use.

## Figures and Tables

**Table 1:** Cohort characteristics.

Characteristic	Overall DSC Cohort	TP	TRC	*p* Value (TP vs. TRC)
Patients	168	139	29	
DSC examinations	224	173 (77.2%)	51 (22.8%)	
Age, years, mean ± SD	56.3 ± 13.1	57.7 ± 12.3	49.2 ± 14.7	0.006
Male	123 (73.2%)	102 (73.4%)	21 (72.4%)	1.000
Female	45 (26.8%)	37 (26.6%)	8 (27.6%)	

Note: *p* values compare patients with TP with patients with TRC; a patient was assigned to the TP group if any of their examinations was TP. Age was compared using Welch’s t-test. Sex distribution was compared using Fisher’s exact test.

**Table 2:** Patient-level training and held-out test split.

Cohort	Patients	Examinations	TP Patients	TRC Patients	TP Examinations	TRC Examinations
Training/internal validation	109	143	90	19	114	29
Held-out test	59	81	49	10	59	22
Total	168	224	139	29	173	51

**Table 3:** DSC-only model performance: best single classifier versus the locked soft-voting ensemble (patient level; 59 held-out patients: 49 TP, 10 TRC).

Performance Metric	Extra Trees, Best Single Model (95% CI)	Soft-Voting Ensemble (95% CI)
Held-out test AUC	0.802 (0.678–0.898)	0.790 (0.667–0.891)
PR-AUC	0.961 (0.922–0.987)	0.958 (0.915–0.986)
Sensitivity for TP	71.4% (56.7–83.4%)	63.3% (48.3–76.6%)
Specificity for TRC	100.0% (69.2–100.0%)	100.0% (69.2–100.0%)
PPV	100.0% (90.0–100.0%)	100.0% (88.8–100.0%)
NPV	41.7% (22.1–63.4%)	35.7% (18.6–55.9%)
Balanced accuracy	85.7% (79.2–91.7%)	81.6% (74.5–88.1%)
F1 score for TP	83.3% (73.7–90.9%)	77.5% (65.8–86.5%)
MCC	0.546 (0.379–0.706)	0.475 (0.315–0.630)
True pos./true neg./false pos./false neg.	35/10/0/14	31/10/0/18

**Table 4:** DSC-only classifier architecture comparison (patient level; held-out metrics in 59 patients: 49 TP, 10 TRC).

Classifier	Final Features	OOF AUC	Held-Out Test AUC	Sensitivity for TP	Specificity for TRC	Balanced Accuracy	Brier Score
Elastic-Net Logistic *	56	0.833 (0.725–0.924)	0.780 (0.633–0.900)	65.3% (50.4–78.3%)	80.0% (44.4–97.5%)	72.7% (56.8–85.9%)	0.175 (0.122–0.238)
RBF-SVM	56	0.806 (0.690–0.900)	0.808 (0.681–0.909)	67.3% (52.5–80.1%)	90.0% (55.5–99.7%)	78.7% (65.3–88.5%)	0.138 (0.090–0.195)
Extra Trees	163	0.822 (0.710–0.914)	0.802 (0.678–0.898)	71.4% (56.7–83.4%)	100.0% (69.2–100.0%)	85.7% (79.2–91.7%)	0.131 (0.093–0.175)
Random Forest *	36	0.810 (0.699–0.903)	0.839 (0.719–0.929)	75.5% (61.1–86.7%)	80.0% (44.4–97.5%)	77.8% (62.7–90.4%)	0.115 (0.073–0.162)
HistGradientBoosting *	59	0.861 (0.782–0.927)	0.737 (0.596–0.859)	59.2% (44.2–73.0%)	80.0% (44.4–97.5%)	69.6% (53.6–82.7%)	0.197 (0.109–0.294)

Footnote: performance metrics for the DSC-only classifiers, with 95% confidence intervals (exact Clopper–Pearson for sensitivity and specificity; patient-level bootstrap for all other metrics), are reported for each metric. Final Features is the number of features retained after univariate selection and Spearman redundancy filtering. OOF AUC is the patient-level out-of-fold AUC, which was the predefined model-selection criterion; held-out metrics are reported at each model’s own training-derived Youden threshold. An asterisk (*) marks the three classifiers combined in the locked soft-voting ensemble.

**Table 5:** Top 15 permutation-ranked DSC rCBV radiomic features (patient-level AUC decrease in 59 held-out patients).

Rank	Radiomic Feature	Image Filter	Feature Family	Feature Class	Mean Test AUC Decrease
1	Dependence entropy	Wavelet LLL	GLDM	Spatial texture	0.0420
2	Mean	Wavelet LHL	First-order	Intensity	0.0167
3	Maximal correlation coefficient	Wavelet LLL	GLCM	Co-occurrence texture	0.0163
4	Small-area low-gray-level emphasis	Original	GLSZM	Spatial texture	0.0151
5	Mean	Wavelet HHH	First-order	Intensity	0.0147
6	Sphericity	Original	Shape	Morphology	0.0139
7	Large-area high-gray-level emphasis	Wavelet LLL	GLSZM	Spatial texture	0.0114
8	Run entropy	Wavelet HLL	GLRLM	Run-length texture	0.0094
9	Median	Wavelet HHL	First-order	Intensity	0.0069
10	Median	Wavelet LLH	First-order	Intensity	0.0057
11	Correlation	Wavelet HLL	GLCM	Co-occurrence texture	0.0053
12	Surface volume ratio	Original	Shape	Morphology	0.0053
13	Contrast	Wavelet LHH	NGTDM	Neighborhood texture	0.0049
14	Maximal correlation coefficient	LoG 3.0 mm	GLCM	Co-occurrence texture	0.0049
15	Median	Wavelet HLL	First-order	Intensity	0.0041

**Table 6:** Exploratory DSC versus ASL comparison on the shared ASL-available held-out subset.

Comparison	Common Patients	TP/TRC	DSC-Only AUC	ASL-Only AUC	AUC Difference	Paired DeLong *p* Value
DSC CBF ensemble vs. ASL (modality-matched)	36	30/6	0.822	0.711	+0.111	0.524
DSC rCBV ensemble vs. ASL (not matched)	36	30/6	0.772	0.789	−0.017	0.819

## Data Availability

The imaging data analyzed in this study are publicly available from The Cancer Imaging Archive as the UCSD-PTGBM collection (https://www.cancerimagingarchive.net/collection/ucsd-ptgbm/, accessed on 3 June 2026). The analysis code supporting the reported results is available from the corresponding author upon reasonable request.

## Supplementary Materials

The following supporting information can be downloaded at: https://www.mdpi.com/article/10.3390/cancers18213567/s1. Table S1: Pairwise DeLong comparisons of held-out AUC (primary rCBV analysis); Table S2: Out-of-fold-derived threshold rules and their held-out operating characteristics; Table S3: Predefined soft-voting candidates ranked by patient-level out-of-fold AUC; Table S4: Perfusion-map sensitivity analysis; Table S5: Reference standard by confirmation route (224 analyzed examinations); Table S6: Fold-wise stability of feature selection for the three ensemble components (training cohort: 109 patients, 143 examinations); Table S7: Fold-wise selection of the 15 most influential (permutation-ranked) features; Table S8: Segmentation-perturbation reliability of DSC rCBV radiomic features (224 examinations); Table S9: Label-permutation overfitting control (training cohort: 109 patients, 143 examinations; internal cross-validation).

## Abbreviations

ASL, arterial spin labeling; AUC, area under the receiver operating characteristic curve; CBF, cerebral blood flow; CI, confidence interval; DSC, dynamic susceptibility contrast; GBM, glioblastoma; GLCM, gray-level co-occurrence matrix; GLDM, gray-level dependence matrix; GLRLM, gray-level run-length matrix; GLSZM, gray-level size-zone matrix; IBSI, Image Biomarker Standardization Initiative; ICC, intraclass correlation coefficient; LoG, Laplacian of Gaussian; MCC, Matthews correlation coefficient; NGTDM, neighboring gray-tone difference matrix; NPV, negative predictive value; PPV, positive predictive value; PR-AUC, precision–recall area under the curve; RANO, Response Assessment in Neuro-Oncology; RBF-SVM, radial-basis-function support vector machine; rCBV, relative cerebral blood volume; SVD, singular value decomposition; TP, tumor-positive disease; TRC, treatment-related change.
