# Supplementary material for: Explainable DSC Perfusion Radiomics for Post-Treatment Glioblastoma Assessment Using a Soft-Voting Machine-Learning Ensemble
Source: Cancers (Basel). 2026 Nov 6;18(21):3567. doi: 10.3390/cancers18213567 (PMC13761451; doi:10.3390/cancers18213567)
Supplement: Supplementary file 1 [file cancers-18-03567-s001.zip › cancers-4623820-supplementary.pdf]

Supplementary Material

Supplementary Table S1. Pairwise DeLong comparisons of held-out AUC (primary rCBV analysis)

Comparison	AUC (Ensemble)	AUC (Individual Model)	ΔAUC	95% CI	DeLong p
Ensemble vs. Elastic-Net Logistic	0.790	0.780	+0.010	-0.100 to +0.120	0.855
Ensemble vs. RBF-SVM	0.790	0.808	-0.018	-0.092 to +0.055	0.624
Ensemble vs. Extra Trees	0.790	0.802	-0.012	-0.070 to +0.046	0.680
Ensemble vs. Random Forest	0.790	0.839	-0.049	-0.125 to +0.027	0.207
Ensemble vs. HistGradientBoosting	0.790	0.737	+0.053	-0.034 to +0.140	0.231

Footnote: ΔAUC = AUC(ensemble) − AUC(model). Positive values favor the ensemble. P-values from paired DeLong tests on the held-out test set (n = 59 patients; 49 TP, 10 TRC). No comparison reached statistical significance. CI = confidence interval. AUCs are patient-level; no comparison was statistically significant (all p > 0.20), which does not establish equivalence.

Supplementary Table S2. Out-of-fold-derived threshold rules and their held-out operating characteristics

Threshold rule (defined on out-of-fold data)	Thr.	OOF Sens.	OOF Spec.	Test Sens.	Test Spec.	Test Bal. Acc.	Test MCC	True pos./true neg./false pos./false neg.
Youden index on OOF (current prespecified primary)	0.811	71.1%	84.2%	63.3%	100.0%	81.6%	0.475	31/10/0/18
OOF sensitivity >= 0.99	0.180	100.0%	5.3%	100.0%	0.0%	50.0%	0.000	49/0/10/0
OOF sensitivity >= 0.95	0.457	96.7%	36.8%	89.8%	10.0%	49.9%	-0.003	44/1/9/5
OOF sensitivity >= 0.90	0.593	91.1%	52.6%	81.6%	30.0%	55.8%	0.108	40/3/7/9
OOF sensitivity >= 0.85	0.633	85.6%	63.2%	79.6%	50.0%	64.8%	0.255	39/5/5/10
OOF sensitivity >= 0.80	0.671	82.2%	68.4%	75.5%	60.0%	67.8%	0.289	37/6/4/12
OOF sensitivity >= 0.75	0.728	76.7%	73.7%	69.4%	80.0%	74.7%	0.380	34/8/2/15
OOF specificity >= 0.70 (current secondary)	0.728	76.7%	73.7%	69.4%	80.0%	74.7%	0.380	34/8/2/15
OOF specificity >= 0.60	0.633	85.6%	63.2%	79.6%	50.0%	64.8%	0.255	39/5/5/10

Footnote: Every threshold is derived from out-of-fold (training) predictions only; the held-out columns report what that rule yields on the independent test set. The Youden rule is the predefined primary operating point, and the specificity ≥ 0.70 rule is the predefined higher-sensitivity operating point; the remaining rows are provided to make the sensitivity–specificity trade-off fully visible and were not used for model selection. Out-of-fold columns are patient-level in the training cohort (109 patients, 143 examinations); test columns are patient-level in the held-out cohort (59 patients, 81 examinations; 49 TP, 10 TRC).

Supplementary Table S3. Predefined soft-voting candidates ranked by patient-level out-of-fold AUC

Candidate	Members	Ensemble	OOF AUC	OOF PR-AUC
hist_gradient_boosting	HistGradientBoosting	No	0.8614	0.9700
elastic_net_logistic	Elastic-Net Logistic	No	0.8327	0.9577
mean_top3_oof_ap	HistGradientBoosting + Elastic-Net Logistic + Random Forest	Yes	0.8322	0.9574
weighted_all5_oof_auc	Elastic-Net Logistic + RBF-SVM + Extra Trees + Random Forest + HistGradientBoosting	Yes	0.8316	0.9552
mean_top3_oof_auc	HistGradientBoosting + Elastic-Net Logistic + Extra Trees	Yes	0.8310	0.9552
weighted_top3_oof_auc	HistGradientBoosting + Elastic-Net Logistic + Extra Trees	Yes	0.8298	0.9550
mean_top2_oof_auc	HistGradientBoosting + Elastic-Net Logistic	Yes	0.8281	0.9558
mean_top2_oof_ap	HistGradientBoosting + Elastic-Net Logistic	Yes	0.8281	0.9558
mean_all5	Elastic-Net Logistic + RBF-SVM + Extra Trees + Random Forest + HistGradientBoosting	Yes	0.8281	0.9546
mean_tree3_extra_random_hgb	Extra Trees + Random Forest + HistGradientBoosting	Yes	0.8257	0.9523
extra_trees	Extra Trees	No	0.8216	0.9503
mean_tree2_extra_random	Extra Trees + Random Forest	Yes	0.8181	0.9503
mean_nonlinear3_rbf_extra_random	RBF-SVM + Extra Trees + Random Forest	Yes	0.8170	0.9496
random_forest	Random Forest	No	0.8099	0.9509
rbf_svm	RBF-SVM	No	0.8064	0.9507

Footnote: The candidate set was fixed before any held-out evaluation. The primary model is the ensemble with the highest patient-level out-of-fold AUC (mean_top3_oof_ap: HistGradientBoosting + Elastic-Net Logistic + Random Forest). Single classifiers are listed as comparators and were not eligible as the primary model, since the study evaluates a soft-voting ensemble. No held-out test metric contributed to this ranking. Out-of-fold AUCs are patient-level in the training cohort (109 patients, 143 examinations).

Supplementary Table S4. Perfusion-map sensitivity analysis

Perfusion map	OOF AUC	Test AUC	95% CI	Thr.	Sens. TP	Spec. TRC	Bal. Acc.	MCC	True pos./true neg./false pos./false neg.
Leakage-corrected CBV (primary)	0.832	0.790	0.667-0.891	0.811	63.3%	100.0%	81.6%	0.475	31/10/0/18
SVD-deconvolved CBF (secondary)	0.820	0.782	0.632-0.900	0.666	75.5%	80.0%	77.8%	0.440	37/8/2/12

Paired comparison across the identical 59 held-out patients: ΔAUC +0.0082 (95% CI -0.107 to +0.124), DeLong p = 0.890.

Footnote: Both arms use the same cohort, the same locked patient-level split, the same feature-extraction settings, the same 200-trial optimization and the same predefined ensemble-selection rule; they differ only in the perfusion map from which features were extracted. The selection rule chose different classifier members for each map (rCBV: HistGradientBoosting + Elastic-Net Logistic + Random Forest; CBF: RBF-SVM + Extra Trees + Random Forest). Thresholds are each model's own out-of-fold Youden value. Test metrics are patient-level (59 held-out patients, 81 examinations). The non-significant AUC difference does not establish equivalence between the two maps.

Supplementary Table S5. Reference standard by confirmation route (224 analyzed examinations)

Class	Confirmation route	Examinations, n (%)	Histopathologic confirmation
TP (n = 173)	Pathology-proven	26–45 (15.0–26.0%)	Yes
TP (n = 173)	Clinical and imaging follow-up	128–147 (74.0–85.0%)	No
TRC (n = 51)	Biopsy-proven pseudoprogression	6 (11.8%)	Yes
TRC (n = 51)	Clinically confirmed pseudoprogression	9 (17.6%)	No
TRC (n = 51)	Radiation necrosis	7 (13.7%)	No (clinically confirmed per descriptor)
TRC (n = 51)	Non-specific post-treatment change	29 (56.9%)	No
All (n = 224)	Histopathologically confirmed	32–51 (14.3–22.8%)	—

Footnote: The data descriptor reports 45 pathology-proven time points among the 192 tumor-positive time points of the source collection but does not release a per-examination flag; because all 19 excluded examinations were tumor-positive, between 26 and 45 of the 173 retained TP examinations were pathology-proven. TRC counts are taken from the released per-examination labels (the descriptor text lists 4 biopsy-proven pseudoprogression, 9 clinically confirmed pseudoprogression, 10 clinically confirmed radiation necrosis and 28 non-specific changes). In the held-out test set, 2 TRC patients had biopsy-proven pseudoprogression and 1 had radiation necrosis; the remaining 7 had non-specific post-treatment change. TP, tumor-positive disease; TRC, treatment-related change.

Supplementary Table S6. Fold-wise stability of feature selection for the three ensemble components (training cohort: 109 patients, 143 examinations)

Component	Top-K	Spearman cutoff	Final features (full training set)	Features per fold, range	Pairwise Jaccard, mean (range)	Chance Jaccard	Final features selected in ≥3/5 folds	Final features selected in 5/5 folds
HistGradientBoosting	1223	0.7	59	52-60	0.81 (0.74-0.87)	0.024	98.3%	69.5%
Elastic-net logistic	300	0.85	56	47-61	0.40 (0.26-0.52)	0.021	75.0%	30.4%
Random Forest	500	0.75	36	34-44	0.41 (0.28-0.58)	0.017	80.6%	38.9%

Footnote: The selection steps of each component (median imputation, variance filtering, ANOVA F-test top-K ranking and Spearman redundancy filtering at the locked settings) were repeated in the training portion of each of the five patient-grouped internal cross-validation folds (StratifiedGroupKFold, seed 42). Applied to the full training set, the same steps reproduce the 59, 56 and 36 features reported in the main text. Chance Jaccard is the mean overlap of random feature sets of the same sizes drawn from the 1,223-feature pool (2,000 draws). Because the Spearman filter keeps one feature from each highly correlated group, a correlated substitute selected in another fold counts as a mismatch, so the Jaccard index is a conservative measure of stability. Across folds, the

standard deviation of each feature family's share of the selected features ranged from 0.5 to 8.2 percentage points (mean 1.5, 3.5 and 2.6 percentage points for HistGradientBoosting, elastic-net logistic regression and Random Forest, respectively).

Supplementary Table S7. Fold-wise selection of the 15 most influential (permutation-ranked) features

Rank	Feature	HistGradientBoosting	Elastic-net logistic	Random Forest	Folds selected by any component
1	wavelet-LLL_gldm_DependenceEntropy	5/5*	4/5*	5/5*	5/5
2	wavelet-LHL_firstorder_Mean	5/5*	5/5*	5/5*	5/5
3	wavelet-LLL_glcm_MCC	5/5*	5/5*	5/5*	5/5
4	original_glszm_SmallAreaLowGrayLevelEmphasis	0/5	5/5*	3/5*	5/5
5	wavelet-HHH_firstorder_Mean	5/5*	0/5	0/5	5/5
6	original_shape_Sphericity	5/5*	3/5*	5/5*	5/5
7	wavelet-LLL_glszm_LargeAreaHighGrayLevelEmphasis	0/5	5/5*	3/5*	5/5
8	wavelet-HLL_glrlm_RunEntropy	0/5	3/5*	5/5*	5/5
9	wavelet-HHL_firstorder_Median	4/5*	0/5	0/5	4/5
10	wavelet-LLH_firstorder_Median	4/5*	0/5	0/5	4/5
11	wavelet-HLL_glcm_Correlation	5/5*	4/5*	5/5*	5/5
12	original_shape_SurfaceVolumeRatio	3/5*	5/5*	5/5*	5/5
13	wavelet-LHH_ngtdm_Contrast	5/5*	1/5	1/5	5/5
14	log-sigma-3-0-mm-3D_glcm_MCC	4/5*	5/5*	5/5*	5/5
15	wavelet-HLL_firstorder_Median	5/5*	0/5	0/5	5/5

Footnote: Entries give the number of the five internal cross-validation training folds in which the feature was selected by each component; an asterisk (*) marks features retained by that component in the final model fitted on the full training set. Rank follows Table 5 of the main text.

Supplementary Table S8. Segmentation-perturbation reliability of DSC rCBV radiomic features (224 examinations)

Feature set	n	ICC(3,1), median (IQR), ±1 and ±2 mm	ICC > 0.90	ICC ≥ 0.75	ICC < 0.50	ICC(3,1), median, ±1 mm only	ICC ≥ 0.75, ±1 mm only
All features	1223	0.90 (0.77–0.94)	49.8%	75.9%	7.9%	0.94	84.4%
HistGradientBoosting features	59	0.70 (0.47–0.85)	16.9%	39.0%	27.1%	0.79	61.0%
Elastic-net logistic features	56	0.83 (0.74–0.88)	19.6%	69.6%	7.1%	0.91	87.5%
Random Forest features	36	0.81 (0.67–0.90)	25.0%	58.3%	16.7%	0.89	80.6%
All ensemble features (union)	108	0.78 (0.59–0.86)	17.6%	54.6%	19.4%	0.87	72.2%
Family: First-order	234	0.92 (0.78–0.95)	59.4%	77.8%	6.8%	—	—
Family: GLCM	312	0.93 (0.84–0.95)	61.5%	84.6%	2.6%	—	—
Family: GLDM	182	0.91 (0.68–0.94)	52.2%	73.1%	11.5%	—	—
Family: GLRLM	208	0.91 (0.82–0.94)	52.9%	76.0%	10.1%	—	—
Family: GLSZM	208	0.84 (0.68–0.90)	26.4%	68.8%	9.1%	—	—
Family: NGTDM	65	0.79 (0.52–0.87)	12.3%	53.8%	18.5%	—	—
Family: Shape	14	0.94 (0.89–0.96)	71.4%	92.9%	0.0%	—	—
Image type: Original	107	0.92 (0.89–0.94)	67.3%	98.1%	0.0%	—	—
Image type: LoG-filtered	372	0.91 (0.84–0.94)	54.8%	85.8%	4.0%	—	—

Image type: Wavelet-filtered	744	0.89 (0.67–0.94)	44.8%	67.7%	11.0%	—	—

Rank	Feature	ICC(3,1), ±1 and ±2 mm	ICC(3,1), ±1 mm only
1	wavelet-LLL_gldm_DependenceEntropy	0.93	0.97
2	wavelet-LHL_firstorder_Mean	0.62	0.75
3	wavelet-LLL_glcm_MCC	0.74	0.76
4	original_glszm_SmallAreaLowGrayLevelEmphasis	0.83	0.91
5	wavelet-HHH_firstorder_Mean	-0.05	-0.07
6	original_shape_Sphericity	0.93	0.96
7	wavelet-LLL_glszm_LargeAreaHighGrayLevelEmphasis	0.84	0.96
8	wavelet-HLL_glrlm_RunEntropy	0.84	0.92
9	wavelet-HHL_firstorder_Median	0.61	0.78
10	wavelet-LLH_firstorder_Median	0.70	0.89
11	wavelet-HLL_glcm_Correlation	0.80	0.88
12	original_shape_SurfaceVolumeRatio	0.74	0.85
13	wavelet-LHH_ngtdm_Contrast	0.21	0.72
14	log-sigma-3-0-mm-3D_glcm_MCC	0.73	0.82
15	wavelet-HLL_firstorder_Median	0.30	0.70

Footnote: Each whole-lesion mask was eroded and dilated by 1 mm and 2 mm (spherical kernel); median lesion-volume changes were −29% (erosion 1 mm), +32% (dilation 1 mm), −49% (erosion 2 mm) and +60% (dilation 2 mm). Features were re-extracted from the leakage-corrected rCBV map with the settings used for the main analysis, including within-mask z-score normalization, so the perturbation also changes the normalization statistics; the original-mask extraction reproduced the stored features (1,219 of 1,223 within 0.01% relative difference; the remainder differed by floating-point amounts on near-zero values). ICC(3,1): two-way mixed-effects, consistency, single-measure intraclass correlation across the five mask conditions; < 0.50 poor, 0.50–0.75 moderate, 0.75–0.90 good, > 0.90 excellent. First-order mean and median features of high-frequency wavelet bands take values close to zero and were the least robust. LoG, Laplacian of Gaussian; GLCM, gray-level co-occurrence matrix; GLDM, gray-level dependence matrix; GLRLM, gray-level run-length matrix; GLSZM, gray-level size-zone matrix; NGTDM, neighboring gray-tone difference matrix.

Supplementary Table S9. Label-permutation overfitting control (training cohort: 109 patients, 143 examinations; internal cross-validation)

Analysis	Ensemble internal patient-level AUC
True labels, fixed pipeline (ensemble)	0.819
HistGradientBoosting / elastic-net logistic / Random Forest	0.860 / 0.833 / 0.794
Permuted labels (200 permutations), mean ± SD	0.532 ± 0.093
Permuted labels, median (95th percentile; maximum)	0.544 (0.686; 0.800)
Permutation p value	0.005

Footnote: Training labels were randomly permuted 200 times, and for each permutation the full patient-grouped 5-fold cross-validated pipeline (median imputation, variance filtering, ANOVA top-K, Spearman filtering, standardization and classifier fitting with balanced sample weights) was re-run with selection settings and classifier hyperparameters fixed; patient-level probabilities were the maximum across examinations, averaged across the three components. Hyperparameters were fixed rather than re-optimized for each permutation. The HistGradientBoosting and elastic-net hyperparameters are those of the locked optimization; for Random Forest, the best optimization trial at the locked selection settings (top-K 500, $|\rho| < 0.75$) was used, so the true-label ensemble AUC (0.819) differs slightly from the locked ensemble (0.832). p = (1 + number of permutations with AUC ≥ observed)/(1 + 200).
